# Relapsing Polychondritis Presenting With Diffuse Tracheal Inflammation

**DOI:** 10.1002/rcr2.70747

**Published:** 2026-08-27

**Authors:** Daigo Imasato, Kazutoshi Toriyama, Hayato Kinoshita, Yukihisa Takeda, Yuko Iwata, Masayuki Kitahara, Hiroshi Kobayashi, Kenji Tsushima

**Affiliations:** ^1^ Department of Respiratory Medicine Tokyo Medical University Hachioji Medical Center Hachioji‐shi Tokyo Japan; ^2^ Department of Rheumatology Tokyo Medical University Hachioji Medical Center Hachioji‐shi Tokyo Japan

**Keywords:** bronchoscopy, relapsing polychondritis, tracheal wall thickening

## Abstract

A 59‐year‐old man presented with sore throat and cough, and chest computed tomography and bronchoscopy revealed airway stenosis. Four McAdam's criteria supported the diagnosis of relapsing polychondritis. High‐dose prednisolone and cyclophosphamide improved symptoms and airway lesions, highlighting the importance of early recognition and serial airway evaluation.

## Graphical abstract

A 59‐year‐old man presented with sore throat and cough, and imaging revealed airway stenosis. Four McAdam criteria confirmed relapsing polychondritis. High‐dose prednisolone and cyclophosphamide improved symptoms and airway lesions.

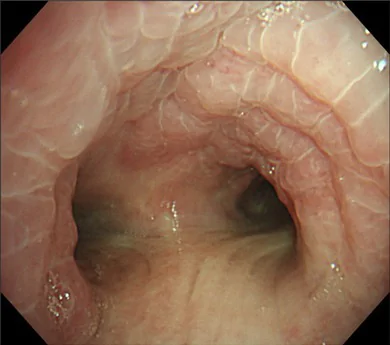

A 59‐year‐old man receiving prednisolone 3 mg/day for polymyalgia rheumatica presented with sore throat and cough. Laboratory tests showed a white blood cell count of 8750/μL and a C‐reactive protein level of 14.2 mg/dL. Chest computed tomography demonstrated diffuse thickening of the tracheal wall (Figure 1A). Bronchoscopy revealed oedematous mucosa along the cartilaginous portion of the trachea (Figure 1B). Although auricular cartilage and bronchial mucosal biopsies were nondiagnostic, the presence of nonerosive inflammatory polyarthritis, nasal chondritis, conjunctivitis and respiratory tract chondritis fulfilled four McAdam's criteria, leading to a clinical diagnosis of relapsing polychondritis (RP). Treatment with prednisolone 40 mg/day and intravenous cyclophosphamide resulted in improvement of symptoms and airway lesions (Figure 1C,D).

**FIGURE 1:**
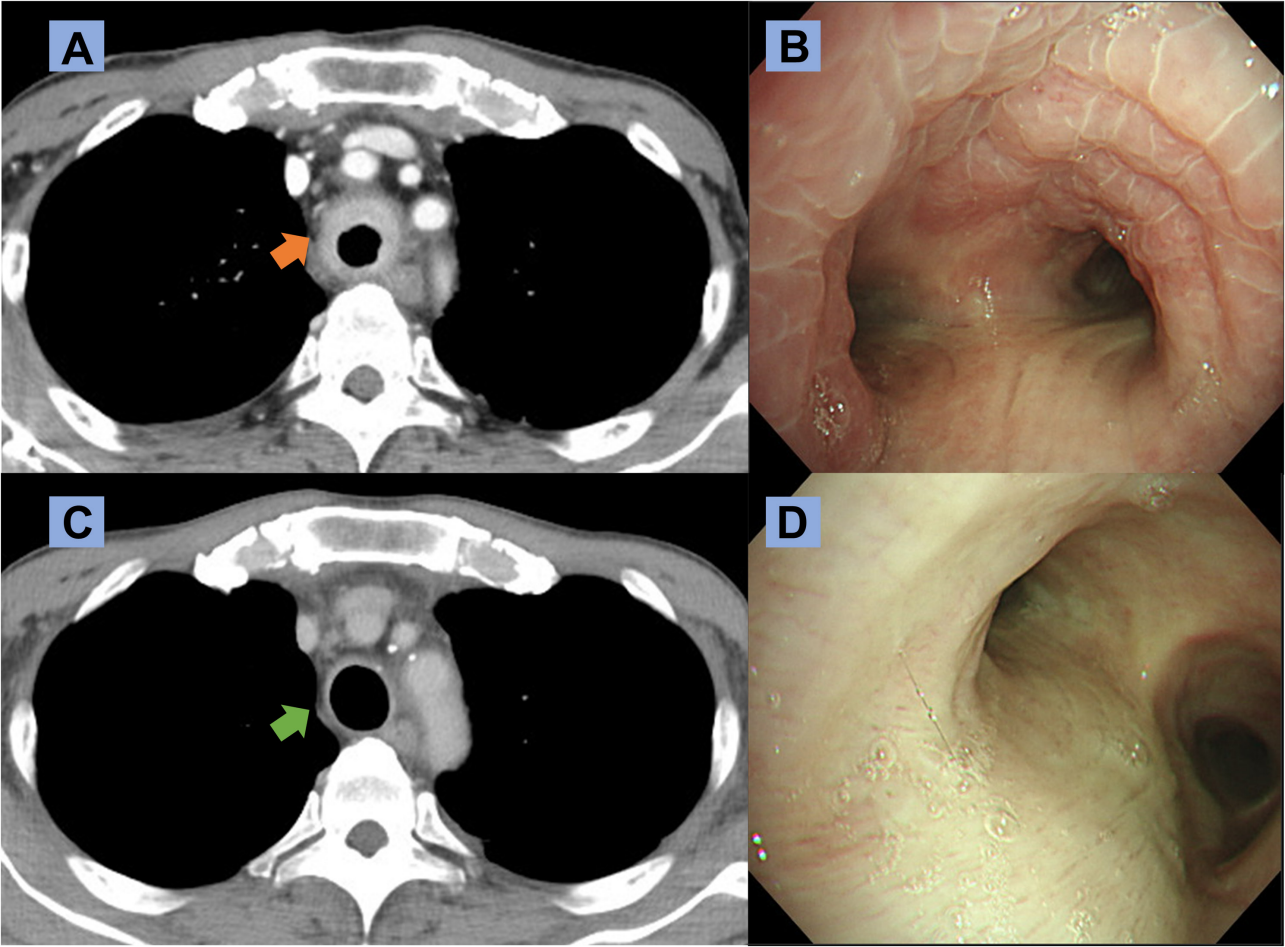
Pre‐treatment chest computed tomography and bronchoscopy show tracheal wall thickening and mucosal oedema (A, B, orange arrow). Post‐treatment imaging demonstrates improvement in tracheal wall thickening and mucosal findings (C, D, green arrow).

RP is a rare systemic disorder that typically affects middle‐aged individuals. Diagnosis is suggested by chondritis of the auricular, nasal, or respiratory tract cartilage, although histopathological confirmation is often not feasible. Because no laboratory test is specific for RP, diagnosis relies on clinical findings and exclusion of alternative conditions [1]. The present case fulfilled four of McAdam's criteria, leading to a clinical diagnosis of relapsing polychondritis [2]. Airway involvement may progress from inflammatory wall thickening and stenosis to irreversible cartilage destruction, making early recognition and serial evaluation with computed tomography and bronchoscopy essential for assessing disease activity and monitoring treatment response.

## Author Contributions

Daigo Imasato and Kazutoshi Toriyama collected the clinical data and drafted the original manuscript. Hayato Kinoshita obtained informed consent from the patient. Yukihisa Takeda, Yuko Iwata, Masayuki Kitahara, Hiroshi Kobayashi and Kenji Tsushima were in charge of patient care and revised the original manuscript. All authors have confirmed the final manuscript and agreed to publication.

## Funding

The authors have nothing to report.

## Consent

The authors declare that written informed consent was obtained for the publication of this manuscript and accompanying images using the consent form provided by the Journal.

## Conflicts of Interest

The authors declare no conflicts of interest.

## Data Availability

Data sharing not applicable to this article as no datasets were generated or analysed during the current study.
